# Model of medical and psychological support for adolescents with depressive behavior

**DOI:** 10.1192/j.eurpsy.2021.1672

**Published:** 2021-08-13

**Authors:** I. Mykhailova, D. Mitelov, T. Matkovska, O. Mayorov, N. Mikhanovska

**Affiliations:** 1 Psychiatry, State Institution “Institute of the Health Care of Children and Adolescents of NAMS of Ukraine”, Kharkiv, Ukraine; 2 Clinical Informatics And Information Technology In Health Care Management, Kharkiv Medical Academy of Postgraduate Education of the Ministry of Health of Ukraine, Kharkiv, Ukraine; 3 Medical, Kharkiv National University, Kharkiv, Ukraine

**Keywords:** depression, adolescent, behavioral disorders

## Abstract

**Introduction:**

Traditional methods of preventing the deviant behavior in adolescents are aimed at clinical variants of certain forms of deviant behavior.

**Objectives:**

The study included 160 teenagers with depression.

**Methods:**

Study design included: Depression Test, Projective Drawing Tests, Pathoharacterological Diagnostic; Package of AUDIT tests for the diagnosis of dependent behavior.

**Results:**

Symptoms of depressive behavior disorder in adolescents depending on age and gender were identified: in girls aged 12-14 – autoaggression, food disorders and suicidal behavior; in boys aged 12-14 – gaming, internet addiction; in boys aged 15-18 – gambling, drug addiction and smoking. Anxiety of younger teenagers turns into a chronic anxiety-dreary depression, with frequent attacks behavioral disorders.

**Conclusions:**

The use of technology makes it possible to identify informative imprinting of stereotypical behavior and the locus of the therapeutic window, provide medical and psychological support for adolescents with depressive disorders and the quality of social functioning, provide primary and secondary prevention of depression progression and the formation of dependent behavior.

**Disclosure:**

No significant relationships.

